# Cerebral Aneurysm Occlusion at 12-Month Follow-Up After Flow-Diverter Treatment: Statistical Modeling for V&V With Real-World Data

**DOI:** 10.3389/fmedt.2021.705003

**Published:** 2021-09-17

**Authors:** Ana Paula Narata, Laura Obradó, Raquel Kalé Moyano, Juan M. Macho, Jordi Blasco, Antonio López Rueda, Luis San Roman, Sebastian Remollo, Claudia Marinelli, Rosana Cepeda, Héctor Fernández, Ignacio Larrabide

**Affiliations:** ^1^CHRU Hôpitaux de Tours, Tours, France; ^2^Neurovascular Unit, Galgo Medical S. L., Barcelona, Spain; ^3^CDI, Hospital Clinic of Barcelona, Barcelona, Spain; ^4^Area de Neurociencias, Hospital Universitari Germans Trias i Pujol, Badalona, Spain; ^5^ECOSISTEMAS—CIC/UNICEN, Tandil, Argentina; ^6^Pladema—CONICET/UNICEN, Tandil, Argentina

**Keywords:** cerebral aneurysms, flow diverter, device sizing, device porosity, time-to-occlusion

## Abstract

**Background:** Flow-Diverter (FD) porosity has been pointed as a critical factor in the occlusion of cerebral aneurysms after treatment.

**Objective:** Verification and Validation of computational models in terms of predictive capacity, relating FD porosity and occlusion after cerebral aneurysms treatment.

**Methods:** Sixty-four aneurysms, with pre-treatment and follow-up images, were considered. Patient demographics and aneurysm morphological information were collected. The computational simulation provided by ANKYRAS provided FD porosity, expansion, and mesh angle. FD occlusion was assessed and recorded from follow-up images. Multiple regression Logit and analysis of covariance (ANCOVA) models were used to model the data with both categorical and continuous models.

**Results:** Occlusion of the aneurysm after 12 months was affected by aneurysm morphology but not by FD mesh morphology. A Time-To-Occlusion (TTO) of 6.92 months on average was observed with an SE of 0.24 months in the aneurysm population surveyed. TTO was estimated with statistical significance from the resulting model for the data examined and was capable of explaining 92% of the data variation.

**Conclusions:** Porosity was found to have the most correction power when assessing TTO, proving its importance in the process of aneurysm occlusion. Still, further Verification and Validation (V&V) of treatment simulation in more extensive, multi-center, and randomized databases is required.

## Introduction

Flow-Diverter (FD) treatment of cerebral aneurysms is a standard in current medical practice for neurovascular treatment (1). Obliteration of the aneurysm after FD treatment was above 73%, with a mean follow-up time ranging from 9.1 to 9.4 months (2). Time-To-Occlusion (TTO), assessed from the time of the intervention until the total angiographic occlusion of the aneurysm observed in follow-up, has shown to be different from case to case. Several clinical and computational studies indicate that the FD choice has an impact on TTO. Various factors affect the occlusion of an aneurysm, ranging from the physiology of the patient, anti-aggregation used, and the aneurysm location to the FD design and morphology (3–5). Understanding under which situations occlusion is favored and providing tools to assess the TTO will improve FD treatment and patient management understanding.

The use of computational stent sizing tools is proving its clinical value (6–9). Accurate stent simulations provide additional information and a rationale for the choice of size and length of the device based on specific patient morphology. So far, the most significant benefit of such tools is the improved accuracy in choosing the proper device size for a particular patient, given by their ability to predict the exact amount of foreshortening of the device before the patient treatment.

Stent porosity to alterations of flow inside the aneurysm was previously studied (10–12). Still, few works have focused on the relation of FD porosity and aneurysm occlusion at follow-up (4, 13). To the best of our knowledge, this is due to the technical difficulty of assessing FD porosity *in vivo*. It is only very recently that imaging modalities and the use of additional radiopaque markers on FDs allowed visualizing individual wires after implantation. Still, FD porosity *in vivo* is a cumbersome and time-consuming task and cannot be performed in a predictive manner by the interventionist (14).

Recent works have described simulation-based methods capable of such assessment *in vivo*, with high accuracy (+95%) and low average error (~3%) compared to the actual device porosity measured *in vivo* (15). In this study, we used device simulation software ANKYRAS (Galgo Medical S. L., Barcelona, Spain) to assess local FD porosity, FD expansion, and FD mesh angle. The main hypothesis driving this study is that the FD morphology of the device implanted in the patient, obtained using computational simulation technology provided by ANKYRAS, will have a statistically significant relationship to the treated aneurysm TTO. Further, from the data studied we approximated a statistical model to estimate TTO based on patient and FD morphology data.

The goal of this study was to assess the use of this new technology as a predictor of aneurysm post-treatment evolution during follow-up, and their mid-long term evolution after treatment. This will help better understanding the model capacities, limitations, and the clinical implications of such models when being used to plan clinical treatment. In this context, the American Society of Mechanical Engineers (ASME) Verification and Validation (V&V) standard has been released, which aims at assessing such models and their proper use in clinical practice (16). Although the standard is not followed in this study, it is the first step toward a standard clinical validation and assessment of the tools used.

## Materials and Methods

In this study, we used imaging data from cerebral aneurysms treated with FDs in three different medical centers in Europe, namely, The University Hospitals of Tours, Toulouse, France, Hospital Clínic de Barcelona, Barcelona, Spain, and Hospital Universitari Germans Trias i Pujol, Badalona, Spain. The institutional review boards of the three institutions approved the data collection and analysis protocol for this study. From the clinical database at each institution, we retrospectively identified cerebral aneurysm treatments using FDs that had a pre-treatment 3D Rotational Angiography (3DRA), a post-treatment angiogram (2D or 3D) with visible FD radiopaque markers and had undergone at least one follow-up catheter angiogram, irrespective of the follow-up time. Follow-up time ranged from 6 to 12 months throughout the sample. A total of 64 aneurysms (*n* = 64) in a meeting of 44 patients, the specified criteria were found. Aneurysm size is reported in Table 1. Intra-procedural and follow-up angiograms were scouted to obtain relevant patient demographics (age and sex), aneurysm, and parent vessel characteristics. Three-dimensional angiographic images were recovered for the 3D model reconstruction of parent vessels and aneurysms at the intervention.

**Table 1:** Statistical descriptive data of sample size and FU time by brand.

**FD**	***N***	**Mean FU time**	**S.E**.	**CV**	**Min**	**Q1**	**Q3**	**Max**
Derivo	8	6.84	0.57	22.14	4.90	9.00	5.10	7.80
P64	8	6.57	0.15	6.00	6.00	7.10	6.10	6.80
Pipeline	26	6.88	0.39	27.11	4.90	11.40	5.80	7.00
Surpass	22	7.21	0.54	27.89	4.70	12.40	6.60	7.50

*CV, coefficient of variation; FD, Flow-Diverter*.

### Image Acquisition and 3D Modeling

Pre-treatment and follow-up anatomical models were generated from 3DRA images using an AXIOM Artis™ (Siemens Medical Solutions, Erlangen, Germany, *n* = 11), an Integris Allura™ system (Philips Healthcare, Best, the Netherlands, *n* = 6), or an INNOVA 3131 IQ™ (General Electric Healthcare, Milwaukee, WI, USA, *n* = 47). Voxel size of 3DRA images ranged from 0.208 mm × 0.208 mm × 0.208 mm to 0.378 mm × 0.378 mm × 0.378 mm. Proximal and distal ends of the FD were localized from 2D or 3D post-treatment sequences, depending on availability, to match the position of the simulated device.

Images were segmented using a threshold-based segmentation. Threshold values were chosen by an expert in neurovascular angiography to best fit the patient's anatomy depending on the contrast dilution density and image quality. When selecting the threshold value, the treated vessel and aneurysm lumen were considered the priority for the reconstruction, since this is the most important feature considered by ANKYRAS simulation when estimating the device fits the anatomy (17). The need for post-processing rate was similar to previous studies, with 13% (8) cases needing additional mesh edition (6, 12). 3D models were then visually validated by expert interventional neuroradiologists (INR). The centerline was computed for the treated branch, and morphological descriptors were calculated along the vessel (18).

### FD Porosity

Porosity was simulated using ANKYRAS software (www.ankyras.com, Galgo Medical S. L., Barcelona, Spain). A thorough explanation of the validation of the porosity simulation is not presented here. That matter deserves a proper study and is currently undergoing. Still, we provide the currently available validation results for the sake of completeness.

The accuracy of porosity simulation was validated by comparing the simulated porosity, which is rendered as a scalar field indicating the ratio of [free surface]/[total surface] at each point of the FD surface, to the porosity measured from the device. Devices were implanted physically (on 3D silicon phantoms) and virtually (on identical digital replicas of the silicon phantoms). The silicon phantoms were scanned in 3D (using 3DRA images), and the porosity of the implanted FD was extracted by manually segmenting each FD wire from the 3DRA images of the silicone models with the deployed FD inside. Simulated porosity, obtained as a scalar field over the surface of the simulated FD, was compared to the porosity measured from the 3D segmentation of FD wires (15, 19). For each wire, its width and length are known, where only the angulation between wires is changed when the FD changes its diameter. This and the total area of the device (obtained using the local perimeter at each cross-section and the separation between contiguous sections) are used to compute the local porosity of the device. To provide a baseline, the porosity obtained from these measurements on the FD deployed on the silicon phantom was compared to the porosity provided by the manufacturer in their product tag.

### Local Porosity Assessment at the Aneurysm Region

**Figure 1:**
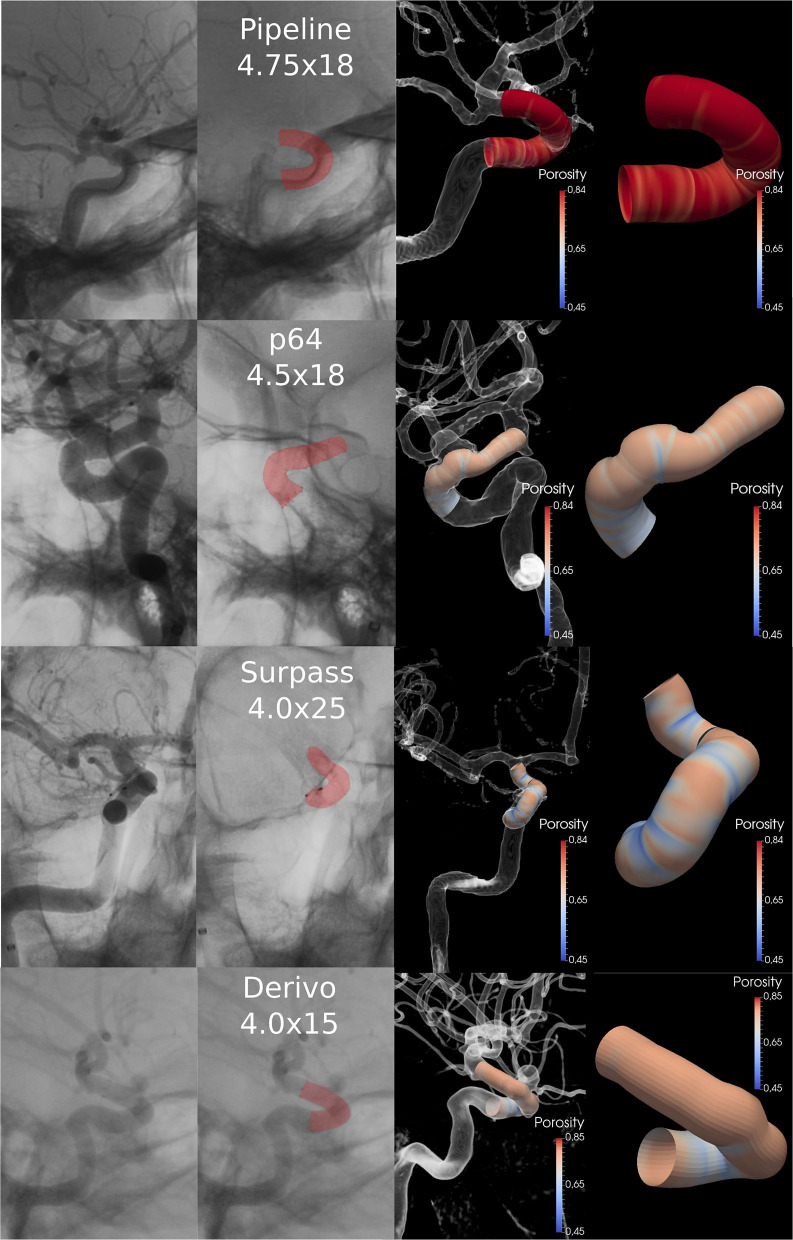
Graphical interface of ANKYRAS, presenting the centerline extraction tools and the quantitative analysis visualization (in this case, cross-section perimeter). The interface of ANKYRAS is 100% hosted by a standard web-browser and can be accessed from any operative system.

**Figure 2:**
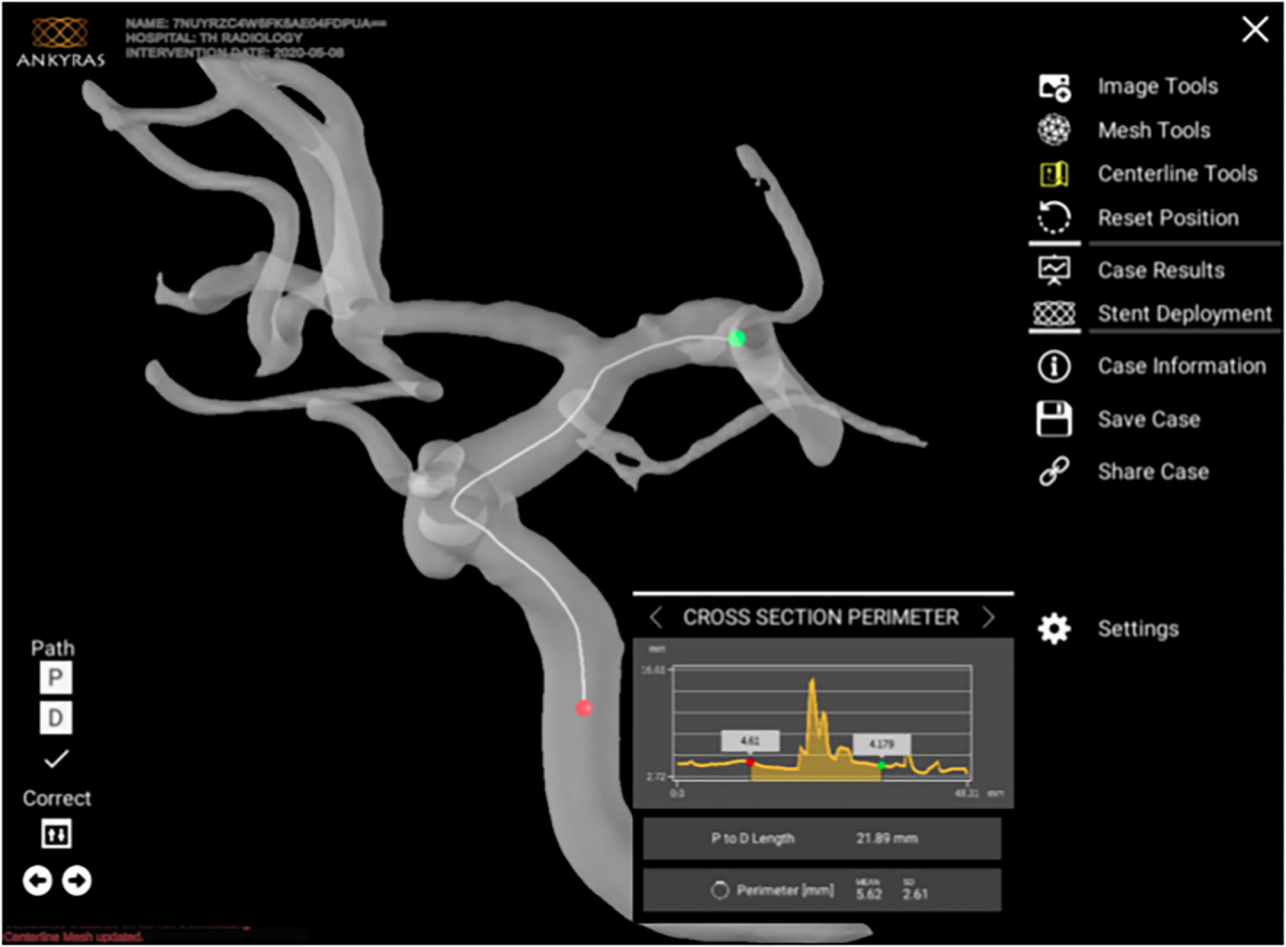
Flow diverter braiding structure, being L the length of the stent at the nominal position and ϕ the nominal diameter of the stent. Right: detailed definition of a unit pore cell, translations and reflections of the unit cell along the surface of the stent covers its whole surface. Being S the constant length of the strut, ω the width of the strut, α the angle with the longitudinal direction of the stent, and S_y_ and S_y_ the projections of the wire at the crossing point. A and B are the non-occupied areas of the unit cell.

For each aneurysm, simulation was done using standard ANKYRAS software workflow. The ANKYRAS software workflow involves segmentation of the 3DRA image, mesh cleaning (to eliminate spurious vessels and noise from the image), centerline extraction of the vessel 3D model, and placement of the device at the location of the aneurysm (Figure 1). The FD is placed by defining the distal position of the implanted device. The same FD brand and size used on the patient were virtually placed on the pre-treatment 3D model, simulating FD length, porosity, and expansion (20). At a glance, the computation of the local device porosity is performed by considering the local braiding angle of the FD wires, the local expansion of the device (the local braiding angle is modified when the expansion changes), and the location of each point on the vessel (the region of the device on the inner side of a vessel curve has a more closed mesh than the region on the outer side). In Figure 2 are presented the design variables that define the local porosity of the FD. These variables are assessed by the algorithm to compute the porosity of the device mesh at each point. Finally, the computation of porosity is extended to the complete FD mesh. We refer the interested reader to (19) for further details on the process of porosity computation. The simulation outputs the porosity, expansion, and mesh angle as a scalar field over the complete mesh of the device. To facilitate the comparison between cases, the average and standard deviation of the FD porosity, mesh angle, and expansion for all the FD points at the aneurysm region were computed for each case, resulting in two values (mean and SD) for each case. The position of the simulated device was defined by identifying the position of the implanted device from post-treatment images and matching the distal position of the device.

### Statistical Data Analysis

Two different analyses were performed on the data. The first one searched for a relation between the FD morphology and the occlusion/patency of the aneurysm at follow-up, such as the whole cohort (occluded and patent aneurysms). In a second analysis, device morphology and TTO were related, only considering occluded aneurysms in the cohort.

Patients and deployed device data analyzed were:

- Patient data: sex and age.
- Aneurysm data: location, depth, volume, width, and maximum and minimum neck diameter.
- Flow diverter morphological data:
  - Mesh porosity: total surface coverage computed as metal covered area/total area.
  - Mesh angle along the longitudinal direction: It is the angle between the FD wires at the crossings using as reference the longitudinal direction of the device. It is measured symmetrically from the longitudinal direction of the FD, i.e., the direction given by the vessel centerline where an angle of 0° corresponds to the stent fully closed, and 180° corresponds to the stent fully open. In practice, both 0° and 180° angles are not reached by the device and are only cited for reference.
  - Mesh expansion: percentage of expansion of the device, being 100% equivalent to the nominal diameter of the device.

The dimensionality of continuous aneurysm morphological data (namely, maximum diameter, minimum diameter, depth, width, and volume) was reduced using Principal Component Analysis (PCA). At a glance, PCA decomposes the data into its principal components (eigenvalues and eigenvectors) containing meaningful information, thus reducing the number of variables required to uphold the main characteristics of the population being observed. The first principal direction was selected as an aneurysm morphology indicator (MAAI), consisting of maximum aneurysm diameter, aneurysm depth, minimum diameter, and neck width as morphological descriptors of the aneurysm. The new variable was recalled as MAAI.

### Occlusion Logit Model

The response to treatment represented by the variable “aneurysm is occluded” was studied with response 0 or 1. The collected data were modeled with a multiple regression Logit model. We hypothesize that if this model is statistically significant, we can state that the variables used by the model influence the aneurysm occlusion in the first 12 months, namely, mesh porosity, mesh angle, mesh expansion, and MAAI.

### Time-To-Occlusion ANCOVA Model

The same four variables plus the FD brand were also studied to determine whether they can explain, in the occluded cases, the different TTO observed. The variable TTO interpretation is “After how many months is likely to see this aneurysm occluded?”. In this case, we considered aneurysms from the previous group occluded at follow-up in the first 12 months following treatment. These variables were considered as fixed factors of the model:

- FD brand (with four levels: Pipeline, Derivo, P64, and Surpass).
- Patient age (in years).

The continuous variables mesh porosity (simulated), mesh angle (simulated), mesh expansion (simulated), and MAAI were included in the model as covariates. Data analysis was performed using the R statistical software package (21).

## Results

**Figure 3:**
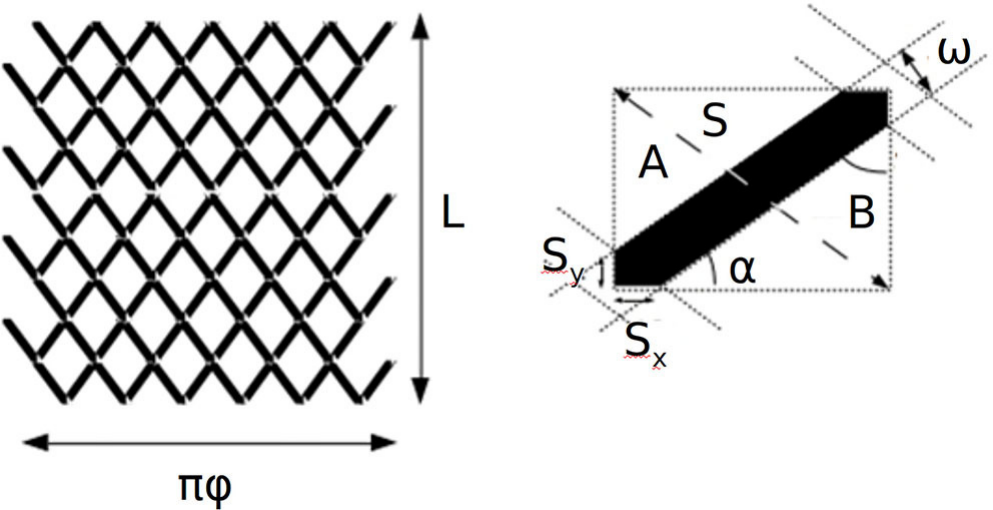
Four sample cases, one for each FD brand used. The first column presents the XA with contrast dilution and the FD. Second column presents the FD highlighted in red. Third column shows the 3D representation of the angiography with the simulated stent implanted at the same distal location as the actual FD. Fourth column shows a close-up of the simulated FD model labeled by porosity. FD, Flow-Diverter.

A total of 44 patients with 64 aneurysms treated with FDs were considered in this study (summarized in Table 1, examples for each brand are presented in Figure 3), namely, 8 (12.5%) patients were treated with DERIVO® Embolization Device (Acandis GmbH & Co, Pforzheim, Germany), 22 (34.4%) were treated with Surpass Streamline™ FD (Stryker Corporation, Kalamazoo, MI, USA), 8 (12.5%) were treated with p64® Flow Modulation Device (Phenox GmbH, Germany), and 26 (40.6%) were treated with Pipeline™ Embolization Device (Medtronic, Minneapolis, MN, USA). Figure 3 shows four sample cases, one for each FD brand used in the study. The number of FDs used for each diameter was 1 FD of 2.50 mm, 2 FDs of 3.00 mm, 5 FDs of 3.5 mm, 32 FDs of 4.00 mm, 6 FDs of 4.25 mm, 11 FDs of 4.50 mm, 6 FDs of 4.75 mm, 1 FD of 5.00 mm, with a total of 64 FDs used.

**Figure 4:**
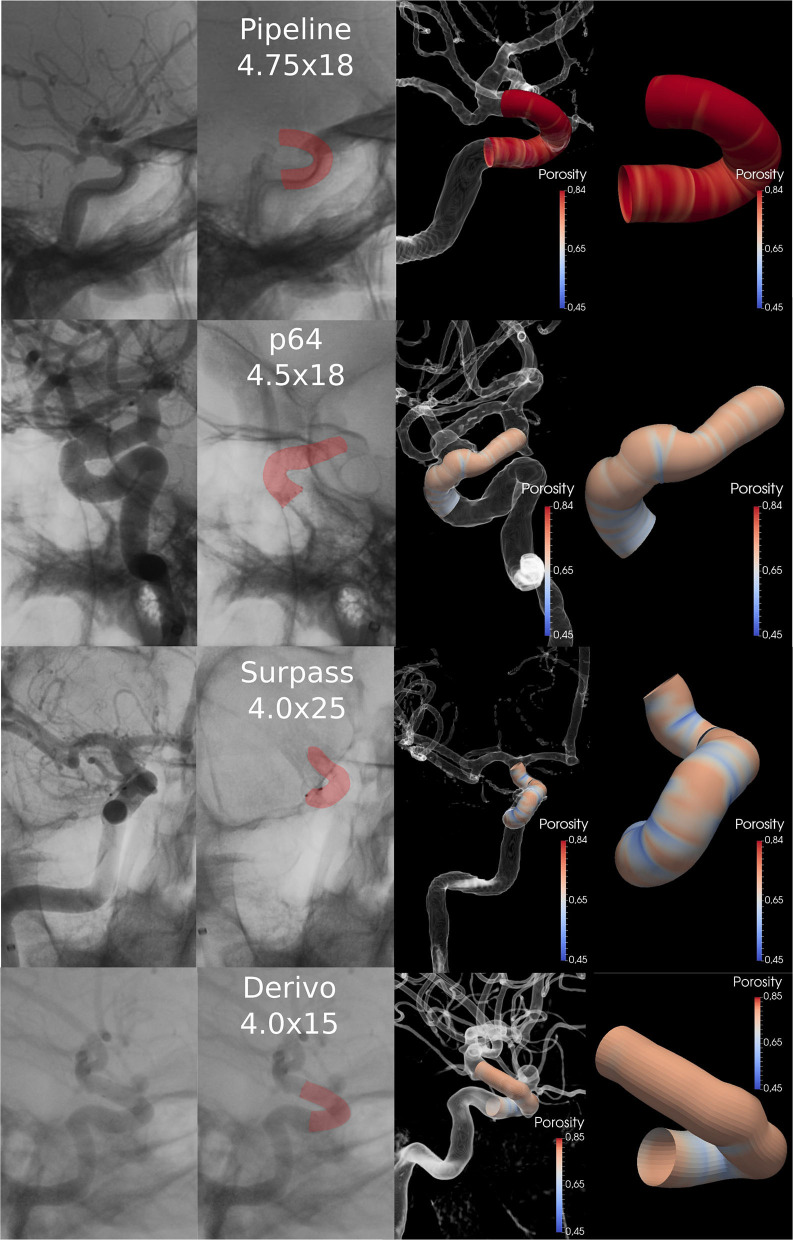
Box-plot of the porosity for each FD, grouped by FD brand. FD, Flow-Diverter.

Aneurysms were located on the internal carotid artery (57), vertebral artery (5), and basilar artery (2). Aneurysms depth was 4.09 mm in average (minimum = 0.73 mm, Q1 = 1.93 mm, median = 3.28 mm, Q3 = 4.97 mm, and maximum = 11.85 mm). MAAI was capable of reconstructing 94% of the total variation in the sample. The average porosity at the treated segment was 72% [standard deviation (STD) 7.4%], average expansion was 87% (STD 7%), and average mesh angle was 101° (STD 13.5 degrees). Figure 4 presents the mean FD porosity for each patient as a box-plot, grouped by FD brand.

The assessment of porosity showed that ANKYRAS porosity simulation estimates the local porosity of the device with improved accuracy compared to the porosity indicated in the product tag provided by the manufacturer, which is obtained from the device nominal expansion. The porosity obtained from the simulation was compared to the former. Simulation of the FD showed 91% of times an error below 10% (considered as a “good estimate”) when compared to the porosity of the implanted device. In the case of porosity estimated following specification of the manufacturers, good estimates were obtained only 58% of the times. This highlights the difference of using ANKYRAS simulation as opposed to using specifications of the manufacturers.

### Logit Regression

Non-significant *p*-values resulted after applying the logit regression for the occlusion-dependent variable when porosity, mesh angle, mesh expansion, MAAI, and FD brand were considered independent variables (porosity *p* = 0.2580, mesh angle *p* = 0.3465, mesh expansion *p* = 0.3664). MAAI showed a *p*-value = 0.075 with a beta coefficient of −0.25. A total of 51 aneurysms were occluded from the 64 observed (13 were still patent after 12 months).

### ANCOVA Analysis

The analysis of covariance (ANCOVA) model requires that data comply with the assumptions of normality and homogeneity of variance of errors, which was the case for our data. The model adjusts very well with *R*^2^ = 0.92 (*p* < 0.0001). The FD brand factor was found to be non-statistically significant. When the factor age was applied, the model was corrected and statistically significant. Still, this correction is not linear since younger and older patients presented similar TTO. In the resulting model, porosity (*p* = 0.0116) and mesh angle (*p* = 0.0994) with a confidence of 90% turned out to be significant as co-variables in the correction.

Age, FD brand, mesh porosity, mesh angle, mesh expansion, and MAAI were used to describe the TTO by an ANCOVA model. The model adjusts very well with *R*^2^ = 0.92 (*p* < 0.0001). This model requires that data comply with the assumptions of normality and homogeneity of variance of errors, which was the case for our data. The FD brand factor was found to be non-statistically significant, Age (*p* < 0.0001), porosity (*p* = 0.0116), and mesh angle (*p* = 0.0994) were significant with a confidence of 95 and 90% for the last one variable. The only variable related to the patient's demographics used by the mode, namely, age, was relevant for the model fit since it explained much of the variability of the data. On the other hand, we pointed out porosity contribution to TTO variation among the relevant variables of the aneurysm, considering the presence of age in the model. Nevertheless, we observed that the age pattern is not linear since younger and older patients presented similar value responses to TTO.

## Discussion

The data were collected in three centers from different countries, using various protocols and four disparate FD brands. The time between treatment and follow-up (FU) varied depending on the patient and the center. Due to the retrospective nature of this study, data heterogeneity in FU was unavoidable. Although the data are heterogeneous, and the number of cases for different brands is not balanced, the overall sample size is sufficient to produce statistically significant results.

The results obtained from the simulation were compared to the porosity indicated by the manufacturers in the product tag. This might be considered as trivial, since we expect personalized simulation to provide a better estimate. Still, this is the only baseline available. We expected simulation to be better, but this remained to be proven. For the population studied, the porosity was higher for Pipeline cases (Figure 4), this might be due to the wire width and number of wires in this device, providing lower metal coverage. This difference did not have an effect on Logit or ANCOVA models, indicating that the particular design, wire width, or arrangement between brands did not have an effect on the occlusion.

FU occlusion at 12 months was first analyzed with a multiple regression Logit model and binary response (occluded or patent). Sixty-four cases entered this sample, where only the morphology MAAI variable had a statistically significant effect on the model (90% CI). Therefore, we can state that MAAI has an incidence of occlusion at FU in the first 12 months. Because of the negative coefficient (beta = −0.25), we can say that smaller aneurysms are more likely to occlude. The rest of the variables did not explain the occlusion. None of the FD mesh-related variables observed (namely, mesh porosity, mesh angle, and mesh expansion) had a statistically significant effect on the response. This means that, from the FD mesh parameters alone, a statistically significant prediction of whether the aneurysm will occlude or not in the first 12 months cannot be drawn. This does not necessarily imply the lack of a relationship between the FD morphology and the occlusion. Conversely, it might be due to the small sample size or due to other effects (such as the use of anti-aggregation, or other patient-related physiological variables not accounted for in this study) that alter the occlusion of the aneurysm. Nevertheless, this approach provides new information, otherwise unavailable, showing the porosity of the device prior to the intervention. From the data presented, we have shown that a link between porosity-related variables and aneurysm occlusion exists, specifically when showing a relation between the TTO (for aneurysms effectively occluded in the first 12 months) and FD porosity. Further studies, including more samples (larger N) and more information for each sample (biological information, more follow-ups, use of anti-aggregation drugs, among others), might provide a better explanation of the dependent variables. Previous results indicate that the effect of FD on intra-aneurysm hemodynamics is substantially different depending on the size of the aneurysm, ultimately leading to occlusion (12, 22). For the analysis performed in the current study, a larger sample might be needed to obtain more conclusive results on this aspect.

A second analysis was run on the data excluding patent aneurysms (i.e., non-occluded) at 12 months from the dataset. On this subset, the time when the aneurysm was observed occluded (TTO) was modeled as the response variable, with FD brand and patient age as factors, and mesh porosity, mesh angle, mesh expansion and aneurysm morphology (MAAI) as correction coefficients. The resulting ANCOVA model showed that patient age had statistically significant predictive value, but not the FD brand. From that, no differences in the response variable TTO were associated with the FD brand, as opposed to patient age, which showed that both younger (between 34 and 47 years) and older (between 53 and 66 years old) patients presented shorter response times than mid-age ones in our cohort. These findings are non-conclusive and deserve further study in a larger cohort.

When looking at co-variables, FD porosity was used as the first *a posteriori* corrector of the model, being the corrected model statistically significant. The interpretation for this is that the porosity is strongly associated with the response variable, TTO. Namely, the predictive power of the ANCOVA model is improved when the porosity variable is incorporated as a corrector to the model. In addition, mesh angle was found to relate to occlusion in the correction of the model, with CI above 90%. It is then not the mesh expansion or the FD brand that affects the TTO, but the porosity itself on the occlusion time during the first 12 months after treatment and, to a lesser extent, the mesh angle. On the other hand, when the correction of the model was done first by aneurysm morphology and mesh angle, the model was also significant. Still, if the model corrected by morphology and mesh angle was then corrected by porosity, only the effect of porosity remained significant, overruling the rest of the correcting variables. Therefore, irrespective of the order that corrections are applied, porosity always has a greater correction power. Again, this shows the importance of FD porosity over TTO. Because similar porosity rates can be obtained for different FD brands and sizes, the simulation might provide valuable information when selecting a device before treatment. For instance, let us assume that simulating a given device produces a low porosity (which we expect to occlude the aneurysm faster), but the device is too long for the segment treated. Simulation and porosity assessment allows testing different devices in the search for a similar porosity yet producing a different FD layout and final length. With porosity simulation, we can assess if a specific porosity can be achieved with more than one device size.

Porosity is a continuous variable, making it an influential co-variable, and not a factor. The porosity does not overrule the rest of the variables but has a strong influence on the TTO model, when this variable is removed; the model is not capable of properly adjusting to the data anymore. The model used is multivariate and, therefore, all the co-variables and factors considered are relevant to the model, which is different from saying that they are significant in the explanation of the response variable (TTO). If a larger N would be available and the model would have more predictive power, all variables would be needed by the model. Still, porosity is not only a co-variable of the model; it is significant in what regards the explanation of the response variable. In summary, we cannot say that porosity is the main factor, but we know its influence and weight on the model is stronger than the other variables.

Occlusion and TTO were modeled using a “black box” approach, based on multiple regression Logit and statistics ANCOVA models. Aneurysm occlusion after FD treatment is mainly driven by obliteration of the aneurysm by the controlled formation of a thrombus inside the sac, produced by flow stasis. Computational modeling of the blood coagulation process is highly complex, involving physiological processes and mechanical interactions between the body and the implanted device (23). The study of this topic has received considerable attention in recent years, but it is not yet possible to produce personalized models that account for all the complexities of blood clotting at the spatial scale of brain arteries and aneurysms (24–26). Personalized blood clotting models are not practical yet, mainly because there is no repeatable way to accurately personalize existing models with patient-specific conditions. The methodology used in this work does not look into the details of the coagulation process. Instead, we observe the predictability of patient and device parameters (model input) on occlusion/patency and TTO (model output) while not considering the underlying processes leading to aneurysm obliteration.

Sample size (*N*) has a significant impact on the statistical significance of the results, which is only achieved if the relationship between the different variables (factors and co-variables) is substantial. Our results indicate that TTO has a strong dependence on patient age but is not directly related to the FD brand. For the age, it was found that younger and older patients present a similar response (TTO), still, this could not be confirmed by previous studies. We consider that this should be carefully analyzed in a dedicated study on a larger sample. The porosity obtained for simulations on patients' anatomy produced a strong correction on the statistical models (ANCOVA) for TTO. This study was performed following a black-box approach, meaning that only a subset of variables was observed. Other variables that might also affect the outcome, such as anti-aggregation, hematocrit, lifestyle, and risk factors, were not individually considered. A single study also reflecting the effect of these variables might provide additional predictive value for modeling occlusion/patency or TTO.

Recent studies related the resistance of the FD mesh covering collateral vessels on their narrowing/occlusion (27). The simulation of FD porosity can become a predictive tool to assess FD resistance, becoming of added value during the selection of FD when collateral vessels are compromised.

The relation of the local mesh configuration and the occlusion of the aneurysm at follow-up is not yet clear. As the FD is more closed (i.e., its diameter is smaller than the nominal diameter), the rhomboidal shapes made by the wires over its surface change their angle, and the local shape of the mesh produced a different porosity locally. The length of the rhomboid sides (i.e., the distance between the individual wires crossings), which might vary between FD brands, also plays a key role in the porosity. These variables were not studied separately by brand mainly because the limited sample size did not ensure the statistical significance of the results. Further, it was not the aim of this work to elaborate on FD design technicalities or on the details of the porosity calculation, which are detailed in the literature. Instead, we focused our research on studying the link between FD porosity-related variables and the occlusion of the aneurysm, not considering the hidden in detail, but as part of a “black box.” Along the same lines, the biological processes behind the aneurysm occlusion were not considered in detail and only the output (occlusion) was linked to the input.

This study aims to assess and evaluate the value and predictive capacity of computational models to treat cerebral aneurysms precisely, to determine the TTO after treatment and its relation to the morphology of the implanted FD. In this study, we found out that although there is a clear relationship between porosity (and related variables) and TTO, our sample is not sufficiently large to produce a model with sufficient predictive value in terms of the time from the treatment to the final occlusion of the aneurysm. Although this might be due to device design, the number of cases was not sufficient to correct for this variability, and different devices were considered to enlarge the sample. Studying the effect of different designs was not the aim of the study. The objective of this study is to evaluate some of the advantages and potential uses of FD simulation in the planning of cerebral aneurysm treatment. In this case, we provide further insights to medical doctors and interventionists on how the information obtained from FD simulation could be interpreted and the context and hypothesis that should be considered for such interpretation. From our results, we have seen that the simulation of FD-derived variables (mesh porosity, mesh angle, and mesh expansion) is correlated to aneurysm occlusion at follow-up. Still further analysis, validation, and data are required to provide solid evidence that can be used in daily clinical practice.

In the future, and aiming to validate cerebral aneurysm treatment simulation tools in the context of the ASME V&V 40 standard, longitudinal studies collecting data from the day of the treatment to several follow-ups might provide the additional evidence needed to strengthen further the FD sizing technology credibility. The combination of pre-treatment simulation with post-treatment follow-up data and imagery will provide a better insight on the efficacy of simulation for treatment outcome prediction, risk mitigation, and reduction of post-operative complications. The guidelines provided by the ASME V&V 40 set out a framework defining methodologies, context, and best practices for this process. First, a proper question of interest will need to be defined. In this context, the question could be the identification of the ideal device size that ensures a safe and early occlusion of an aneurysm. Secondly, a context of use (COU) will need to be defined. This COU will consider the availability of pre-treatment data, available device brands, and sizes at the intervention site, and a post-treatment data acquisition protocol that ensures a sustained follow-up of the treatment. In the third place, model risk will need to be assessed in the context of the patient resulting harmed from the use of FD simulation for sizing. The definition of the model's risk should consider the effect of the simulation output on the interventionist decision-making process (model influence) and the consequence of negative treatment outcomes resulting from inaccurate or incorrect decision-making due to model output (decision consequence). Finally, the definition of credibility factors driven by the risk analysis will provide evaluation tools for the simulation. This work is not yet started for ANKYRAS as a clinical tool, yet is the intention to follow these steps and standards in the near future.

## Limitations

Aneurysm occlusion is a complex phenomenon driven by the mechanics and shape of the aneurysm and the biology of the patient, anti-aggregation drugs, among others. The statistical modeling tools used in this study only consider some of the phenomena and variables as inputs and relates them to the output (occlusion). Using a black-box approach does not imply that we neglect the effect of those variables, just that we do not describe them in detail. Further studies could collect such variables and output a more detailed model.

The fact that the sub-analysis was performed only on the sub-set of aneurysms occluded during the first 12 months might be a source of bias, limiting the generalization of the study. This analysis should be extended to many cases that ensure a precise, more evident tendency in the model.

## Data Availability Statement

The datasets used and analyzed during the current study will be made available by the corresponding author upon reasonable request.

## Ethics Statement

The studies involving human participants were reviewed and approved by The University Hospitals of Tours, Toulouse, France, Hospital Clínic de Barcelona, Barcelona, Spain and Hospital Universitari Germans Trias i Pujol, Badalona, Spain institutional review boards approved the data collection and analysis protocol of this retrospective study. The patients/participants provided their written informed consent to participate in this study.

## Author Contributions

AN, JM, JB, AR, and SR performed data collection. LO, RM, CM, RC, HF, and IL performed the statistical data analysis and figures. AN, HF, and IL performed the manuscript drafting. All authors helped in the revision, drafting of the manuscript, and agreed on the final version of the manuscript.

## Funding

This work was partly funded by ANPCyT PICT 2016-0116 and PICT Start-up 2020-00045, and by MINECO RTC-2016-5073-1 POROVIEW.

## Conflict of Interest

IL and HF hold ownership of stock in Galgo Medical S. L. LO and RK were employed by Galgo Medical S. L. at the time of preparation of the manuscript. The remaining authors declare that the research was conducted in the absence of any commercial or financial relationships that could be construed as a potential conflict of interest.

## Publisher's Note

All claims expressed in this article are solely those of the authors and do not necessarily represent those of their affiliated organizations, or those of the publisher, the editors and the reviewers. Any product that may be evaluated in this article, or claim that may be made by its manufacturer, is not guaranteed or endorsed by the publisher.
